# Proliferative Capacity of Adult Mouse Brain

**DOI:** 10.3390/ijms22073449

**Published:** 2021-03-26

**Authors:** Mikhail Semenov

**Affiliations:** The Department of Pathology and Laboratory Medicine, Boston University School of Medicine, Boston University, Boston, MA 02215, USA; mvs@bu.edu

**Keywords:** adult neurogenesis, neural stem cell, adult brain, brain regeneration, brain repair, cell proliferation, subventricular zone, subgranular zone, neuronal renewal, neuronal replacement

## Abstract

We studied cell proliferation in the postnatal mouse brain between the ages of 2 and 30 months and identified four compartments with different densities of proliferating cells. The first identified compartment corresponds to the postnatal pallial neurogenic (PPN) zone in the telencephalon; the second to the subpallial postnatal neurogenic (SPPN) zone in the telencephalon; the third to the white matter bundles in the telencephalon; and the fourth to all brain parts outside of the other three compartments. We estimated that about 3.4 million new cells, including 0.8 million in the subgranular zone (SGZ) in the hippocampus, are produced in the PPN zone. About 21 million new cells, including 10 million in the subependymal zone (SEZ) in the lateral walls of the lateral ventricle and 2.7 million in the rostral migratory stream (RMS), are produced in the SPPN zone. The third and fourth compartments together produced about 31 million new cells. The analysis of cell proliferation in neurogenic zones shows that postnatal neurogenesis is the direct continuation of developmental neurogenesis in the telencephalon and that adult neurogenesis has characteristics of the late developmental process. As a developmental process, adult neurogenesis supports only compensatory regeneration, which is very inefficient.

## 1. Introduction

The mammalian brain has a very limited capacity for self-repair. For a long time this was attributed to the absence of neurogenesis in the adult mammalian brain [1,2]. However, in the mid-1960s, the first evidence that neurogenesis could be present in the adult mammalian brain had started to appear [3]. The following fifty years brought many exciting discoveries that unequivocally showed the existence of neurogenesis in the adult mammalian brain [4,5]. Toward the mid-1980s, it was established that adult-born neurons have the electrophysiological properties of real neurons [6,7]. At the beginning of the 1990s, cells with properties resembling stem cells were isolated from the adult mouse brain [8,9]. This was followed by the discovery of stem cells in the subependymal zone (SEZ) in the lateral walls of the lateral ventricles [10] and in the subgranular zone (SGZ) in the hippocampus [11].

All these discoveries have debunked the old assumption about the inability of the adult mammalian brain for self-repair due to the absence of neurogenesis. However, the reasons for the very limited capacity for self-repair in the brain become even more puzzling. The presence of stem cells in other adult tissues, such as skin or blood, provides them with a high capacity for self-renewal and repair. Why is this not the case for the brain? In this study, we analyzed the production of new cells in the entire mouse brain between the ages of 2 and 30 months to get insight into the reasons for the insufficient regenerative capability of the adult mammalian brain. 

## 2. Results

**Figure 1:**
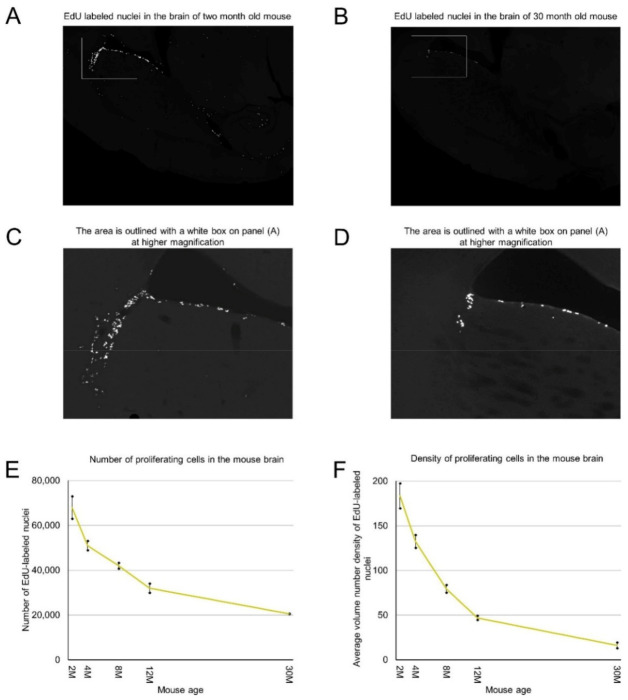
Age-related decrease in the number and density of proliferating cells in the mouse brain. (**A**–**D**) Proliferating cells are distributed in the mouse brain unevenly. 2 months (**A**) and 30 months (**B**) old mice. (**C**) The area outlined with a white box on panel (**A**) is shown at high magnification. (**D**) The area outlined with a white box on panel (**B**) is shown at high magnification. (**E**) The number of detected proliferating cells in the mouse brain decreases with mouse age. The means are significantly heterogeneous (one-way analysis of variance (ANOVA), F4, 5 = 316, *p* = 3.6 × 10^−6^). Error bars show standard deviation. (**F**) The average volume number density of proliferating cells in the mouse brain decreases with mouse age. The means are significantly heterogeneous (one-way ANOVA, F4, 5 = 160, *p* = 31.8 × 10^−5^). Error bars show standard deviation.

We labeled proliferating cells in 2, 4, 8, 12, and 30-month-old mice by intraperitoneal injection of 5-Ethynyl-2′-deoxyuridine (EdU), extracted and processed their brains, and cut them transversely. We stained all sections for each analyzed brain and counted EdU-labeled nuclei of proliferating cells on each section. By summing the numbers of detected proliferating cells on all brain sections we calculated the number of proliferating cells in each analyzed mouse brain (Table 1). We found that mice of the same age have a similar number of proliferating cells (Table 1). Two-month-old mice have about 67,000 proliferating cells. This number decreases steadily with mouse age and in the brains of 30-month-old mice we detected only about 20,000 proliferating cells. The rate of decrease is 12% per month in 2–4-month-old mice and less than 2% in mice over one year old (Figure 1E, Table 1).

**Figure 2:**
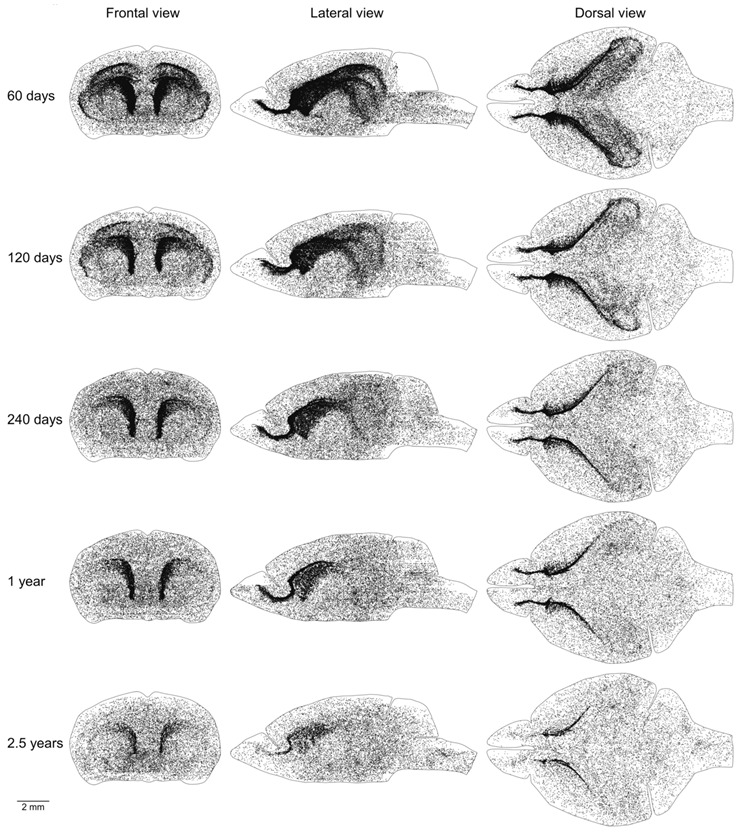
Distribution of proliferating cells in the mouse brain. Each 5-Ethynyl-2′-deoxyuridine (EdU)-labeled nucleus is shown as a black dot. The scale is shown at the bottom. The brain shape is outlined with a black line. Frontal, lateral, and dorsal views of the brain are shown. Mouse age is shown on the left.

Proliferating cells are distributed in the mouse brain unevenly (Figure 1A–C). Therefore, we obtained coordinates for all EdU-labeled nuclei and reconstructed their distribution in all analyzed brains (Figure 2). Proliferating cells are distributed throughout the entire mouse brain with a clearly identifiable aggregation in the middle part of the brain. This aggregation is the most prominent in 2-month-old mice, but its prominence fades with age and in 30-month-old mice, the extent and the density of proliferating cells within the aggregation decreased significantly (Figure 2). The distribution of proliferating cells outside of the aggregation appears to be homogeneous and does not change with mouse age (Figure 2). To characterize the distribution of proliferating cells we calculated the volume number density (mentioned as “density” in the following text) for each EdU-labeled nucleus and found that the average density decreases 10 fold from 180 in the brains of 2-month-old mice to 18 in the brains of 30-month-old mice (Figure 1F, Table 1).

**Figure 3:**
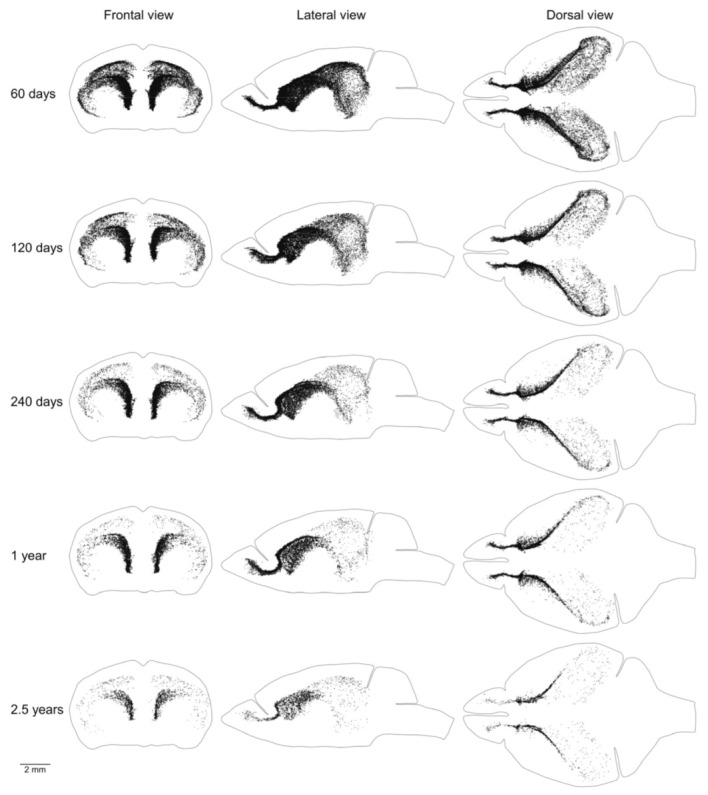
Distribution of proliferating cells in the main proliferative zone (MPZ). Each EdU-labeled nucleus is shown as a black dot. The scale is shown at the bottom. The brain shape is outlined with a black line. Frontal, lateral, and dorsal views of the brain are shown. Mouse age is shown on the left.

**Figure 4:**
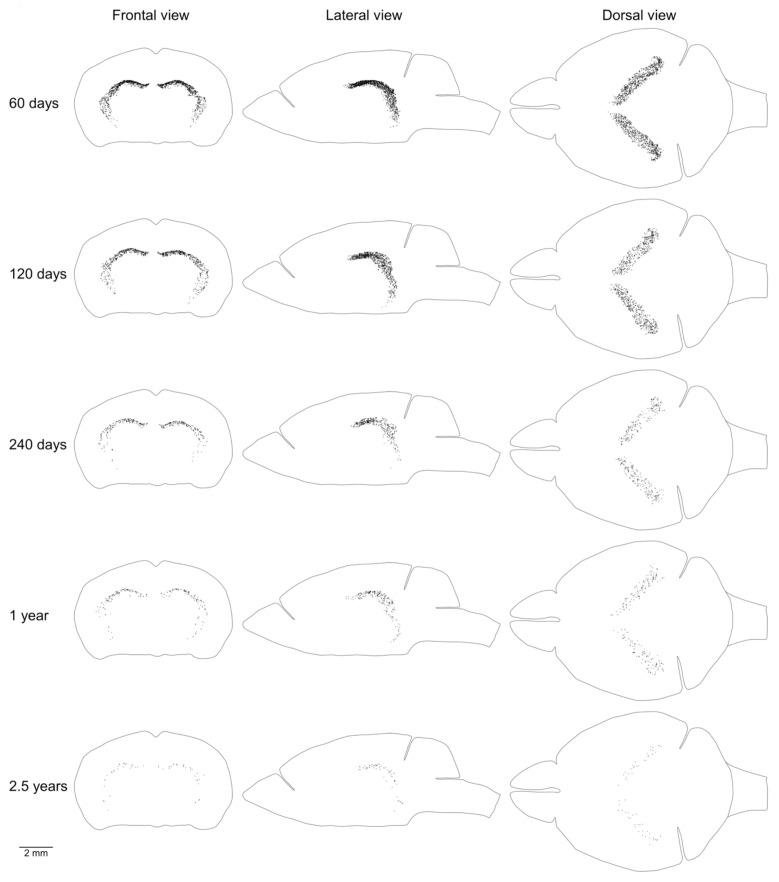
Distribution of proliferating cells in the subgranular zone (SGZ). Each EdU-labeled nucleus is shown as a black dot. The scale is shown at the bottom. The brain shape is outlined with a black line. Frontal, lateral, and dorsal views of the brain are shown. Mouse age is shown on the left.

**Figure 5:**
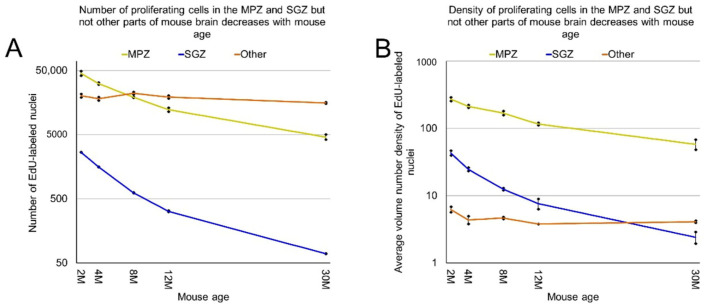
The number and density of proliferating cells in the MPZ, SGZ but not other parts of the mouse brain decrease with mouse age. (**A**) The number of proliferating cells in the MPZ, SGZ, and other parts of the mouse brain changes with mouse age. Error bars show standard deviation; (**B**) the average volume number density of proliferating cells in the MPZ, SGZ, and other parts of the mouse brain changes with mouse age. Error bars show standard deviation.

We showed previously that the aggregation of proliferating cells in the middle of the mouse brain can be divided into two continuums of proliferating cells. One of them is the main proliferative zone (MPZ) and another is the subgranular zone (SGZ) in the hippocampus [12]. We identified proliferating cells located in these continuums and visualized their distribution in the mouse brain (Figure 3 and Figure 4). We also counted the number and density of proliferating cells in each continuum and other parts of the mouse brain (Figure 5, Table 1). The MPZ of 2-month-old mice contains about 45,000 proliferating cells, which account for 65% of all proliferating cells in the mouse brain. This number decreases 10 fold to about 4500 cells in the brains of 30-month old mice, which account for about 23% of all proliferating cells. At the same time, the density of proliferating cells in the MPZ decreases 5 fold from 280 to 65 (Figure 5A,B, Table 1). The SGZ of 2-month-old mice contains about 2600 proliferating cells, which account for less than 4% of all proliferating cells in the mouse brain. With age, this number decreases 40 fold to 70 cells in the brains of 30-month-old mice, which account for less than 0.4% of all proliferating cells. The density of proliferating cells in the SGZ of 2-month-old mice is 45, or about 6 times lower than in the MPZ. The density decreases almost 20 fold to 2.5 in 30-month-old mice (Figure 5A,B, Table 1). The number and density of proliferating cells in other parts of the mouse brain, in contrast with the MPZ and SGZ, remain fairly constant between the ages of 2 and 30 months. The number of proliferating cells decreases only by 25%, from 20,000 to 15,000, and the density is down 35% from about 6 to 4 (Figure 5, Table 1).

**Figure 6:**
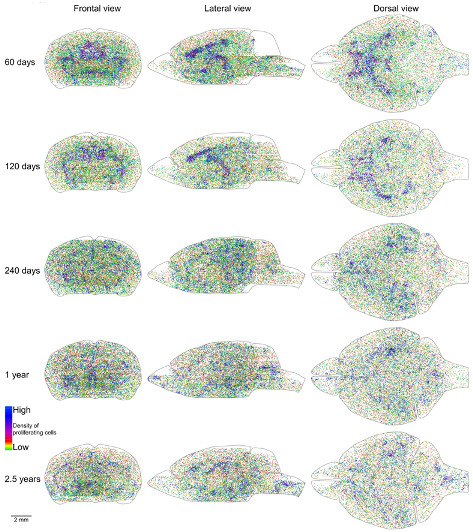
Distribution of proliferating cells outside of the MPZ and SGZ. Each EdU-labeled nucleus is shown as a colored dot with colors assigned according to the number of neighboring EdU-labeled nuclei located closer than 200 µm to this nucleus. The color scale is shown on the bottom left. The brain shape is outlined with a black line. Frontal, lateral, and dorsal views of the brain are shown. Mouse age is shown on the left. The size scale is shown at the bottom.

**Figure 7:**
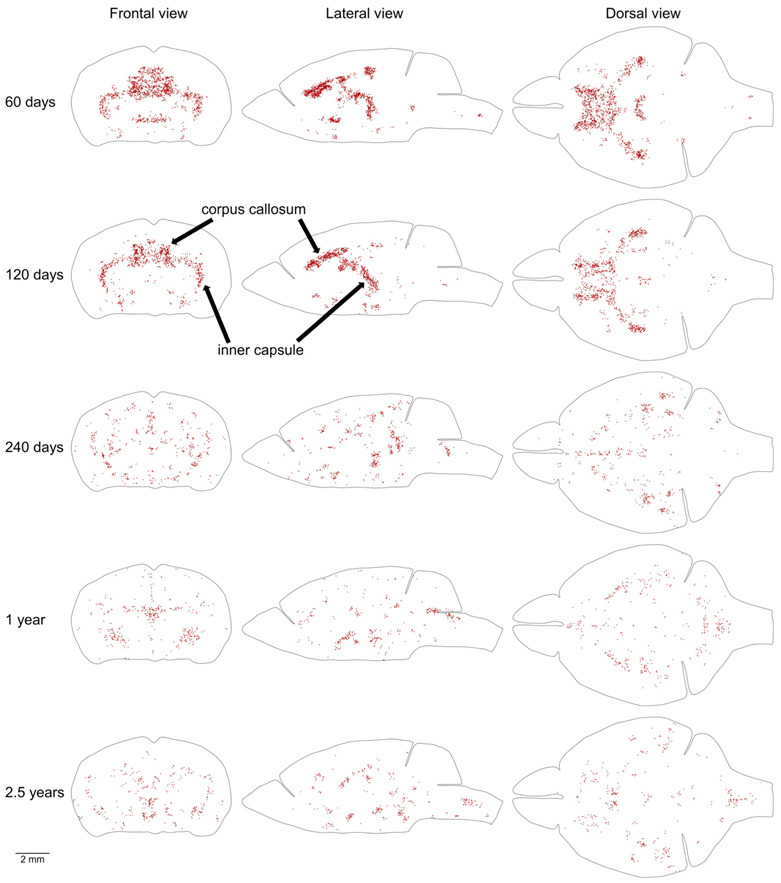
Distribution of proliferating cells in areas with the highest density of proliferating cells outside of the MPZ and SGZ. The distribution of 20% of cells located outside of the MPZ and SGZ that have the highest volume number density is shown. To exclude cells located in the random aggregations of proliferating cells we show only cells in the areas with higher density on both sides of the brain. Each EdU-labeled nucleus is shown as a red dot. The brain shape is outlined with a black line. Frontal, lateral, and dorsal views of the brain are shown. Mouse age is shown on the left. The size scale is shown at the bottom.

Proliferating cells located outside of the MPZ and SGZ are distributed throughout the entire mouse brain with a low density (Table 1, Figure 6). In addition, in the brains of 2 and 4-month-old mice, some areas with a higher density of proliferating cells can be clearly distinguished (Figure 6). We selected 20% of proliferating cells located outside of the MPZ and SGZ with the highest densities in each mouse brain and visualized their distribution in the brain. To exclude random aggregations of proliferating cells we show only cells in the areas with a higher density on both sides of the brain (Figure 7). The most prominent amongst these areas are the inner capsule and the corpus callosum (CC) (Figure 7). The remaining areas of higher density appear to correspond to other white matter tracts in the telencephalon. All these areas of higher density disappear in 8-month-old mice (Figure 7).

**Figure 8:**
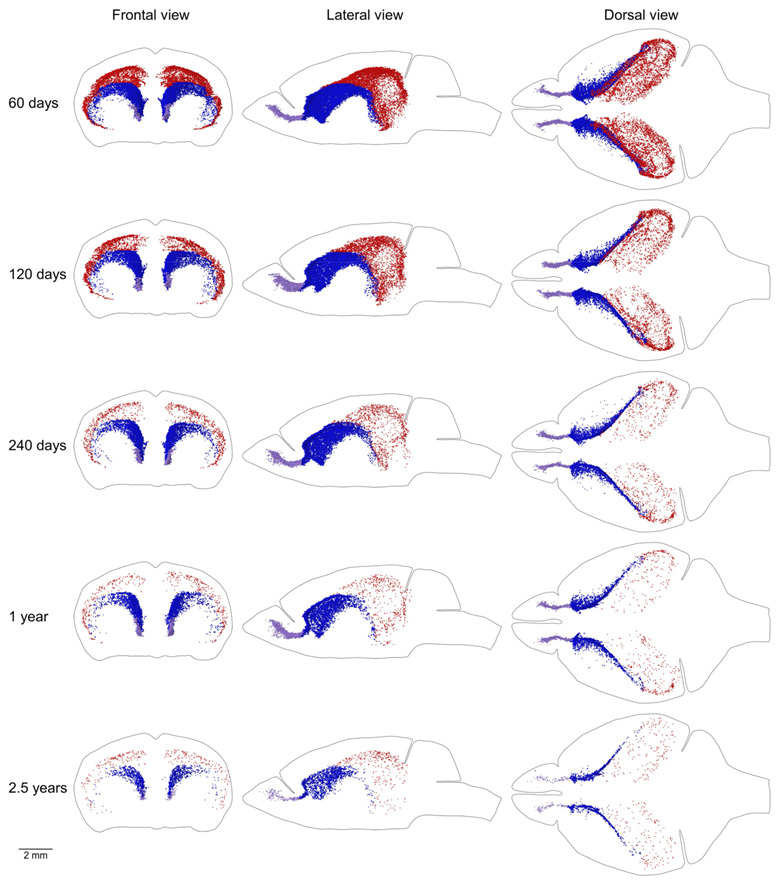
Distribution of proliferating cells in the caudate migratory stream (CMS), midlayer, and rostral migratory stream (RMS). Each EdU-labeled nucleus located in the CMS is shown as a blue dot, in the midlayer as a red dot, and in the RMS as a lilac dot. The scale is shown at the bottom. The brain shape is outlined with a black line. Frontal, lateral, and dorsal views of the brain are shown. Mouse age is shown on the left.

**Figure 9:**
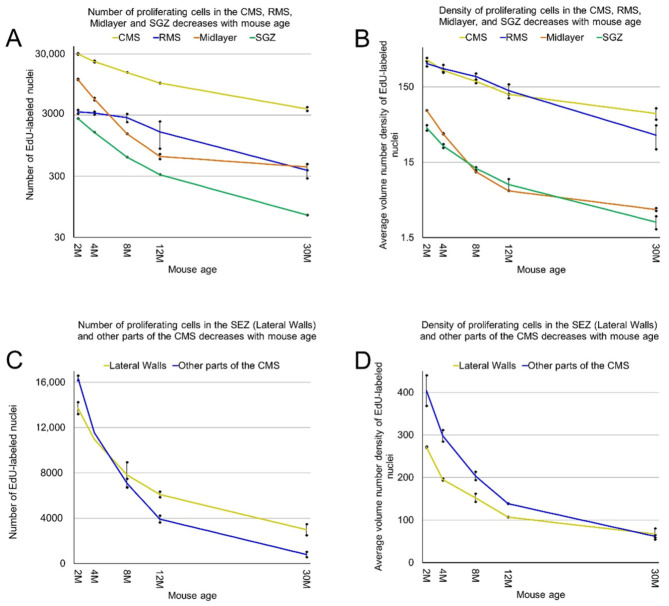
Age-related decrease in the number and density of proliferating cells in the CMS, RMS, midlayer, and SGZ. (**A**) The number of detected proliferating cells in the CMS, RMS, midlayer, and SGZ decreases with mouse age. The means of the number of proliferating cells in the CMS are significantly heterogeneous (one-way ANOVA, F4, 5 = 950, *p* = 2.2 × 10^−7^). The means of the number of proliferating cells in the RMS are significantly heterogeneous (one-way ANOVA, F4, 5 = 19, *p* = 3.1 × 10^−3^). The means of the number of proliferating cells in the midlayer are significantly heterogeneous (one-way ANOVA, F4, 5 = 1783, *p* = 4.5 × 10^−8^). The means of the number of proliferating cells in the SGZ are significantly heterogeneous (one-way ANOVA, F4, 5 = 828, *p* = 3.1 × 10^−7^); (**B**) the average volume number density of detected proliferating cells in the CMS, RMS, midlayer, and SGZ decreases with mouse age; (**C**) The number of detected proliferating cells in the subependymal zone (SEZ) and other parts of the CMS decreases with mouse age; (**D**) the average volume number density of detected proliferating cells in the SEZ and other parts of the CMS decreases with mouse age.

The MPZ can be divided into three parts. The first part is the midlayer that includes proliferating cells located under the external capsule (EC). The second part is the caudate migratory stream (CMS) located in the dorsal and medial walls of the caudoputamen (CP), and the third part is the rostral migratory stream (RMS) connecting the CMS and the olfactory bulbs [12] (Figure 8). The midlayer contains about 11,500 proliferating cells in the brains of 2-month-old mice. The density of these cells is about 75, which is 4.5 times lower than in other parts of the MPZ. The cell number decreases 30 fold to 400 cells and the density 20 fold to about 3.5 in the brains of 30-month-old mice (Figure 9A,B, Table 1). We detected about 30,000 proliferating cells in the CMS of 2-month-old mice, which account for about 45% of all proliferating cells in the brain. The average density of these cells is about 340, the highest among all analyzed areas of the mouse brain. The cell number in the CMS decreases steadily with mouse age and in 30-month-old mice, we detected only 3800 proliferating cells, which account for 20% of all proliferating cells in the brain. At the same time, the density of these cells decreases almost 5 times to 65 and still remains the highest among all analyzed areas (Figure 9A,B, Table 1). The density of proliferating cells in the RMS of 2-month-old mice is similar to the density observed in the CMS but the cell number is 10 times lower (Table 1). The number and density of proliferating cells decrease 9 fold to 380 and 35 correspondingly in the brain of 30-month-old mice (Figure 9A,B, Table 1).

The CMS can be divided into two parts. One of them is the subependymal zone (SEZ), located in the lateral walls of the lateral ventricles. The SEZ is also known as the subventricular zone (SVZ). This SVZ is different from the pallial SVZ in the developing mouse brain [13] and, to avoid confusion, we will use the term SEZ. The SEZ is the established zone of neurogenesis in the postnatal mouse brain. Another part of the CMS is located in the dorsal wall of the CP and has no direct contact with the lateral ventricle [12]. We found that in the brains of 2-month-old mice, only 45% of proliferating cells in the CMS are located in the ventricle walls in the SEZ, and 55% in another part of the CMS. In addition, the density of proliferating cells in the SEZ is lower than in another part, 270 and 400 correspondingly. However, with age, the number of proliferating cells in another part of the CMS decreases faster than in the SEZ, and in 30-month-old mice, we detected only 800 proliferating cells over there compared to 3000 cells in the SEZ. The density of proliferating cells in the SEZ remains lower than the density in another part of the CMS and only in 30-month-old mice they become similar in both parts, about 65 (Table 1).

Using the numbers of proliferating cells detected in the mouse brain at different ages (Table 1) we estimated that about 55 million new cells are produced in the mouse brain between ages 2 and 30 months (Table 2). About 23 million (42%) of them are produced in the MPZ, 0.8 million (1.5%) in the SGZ, and 31 million (56%) in other parts of the mouse brain. The majority of new cells in the MPZ, about 18 million (77%), are produced in the CMS, and about 2.5 million cells (11% each) in the RMS and the midlayer. New cells in the CMS are produced mostly in the lateral walls of the lateral ventricles, about 10 million (57%), and about 8 million (43%) in another part of the CMS (Table 2).

Mice of the same age have very similar numbers of proliferating cells in the brain and its parts (Table 1). The distribution of proliferating cells on the left and right sides of the brain is also very similar (Figure 2, Figure 3 and Figure 4 and Figure 6, Figure 7, Figure 8). Such similarity might be attained only via a very tight regulation of cell proliferation. The difference in density of proliferating cells in various parts of the mouse brain implies that cell proliferation in these parts might be regulated by different molecular mechanisms. Conversely, a similar density, especially if it changes comparably with the mouse aging, should define compartments in the mouse brain with the similar regulation of cell proliferation. We can distinguish four such compartments in the mouse brain. The CMS and RMS form the first compartment with the highest density of proliferating cells among all four compartments, about 300–350, in the brain of 2-month-old mice (Table 1, Figure 8 and Figure 9B). The SGZ and midlayer form the second compartment with the density of proliferating cells about 40–70 in 2-month-old mice (Table 1, Figure 9B). The third compartment is formed by the white matter bundles in the telencephalon that have a density of proliferating cells slightly higher than in the surrounding parts of the brain. This compartment can be distinguished only in the brains of 2 and 4-month-old mice (Figure 7). The fourth compartment is formed by all brain parts outside of the other three compartments. The density of proliferating cells in this compartment is about 4–6 and it does not change with mouse age (Table 1, Figure 5B).

## 3. Discussion

**Figure 10:**
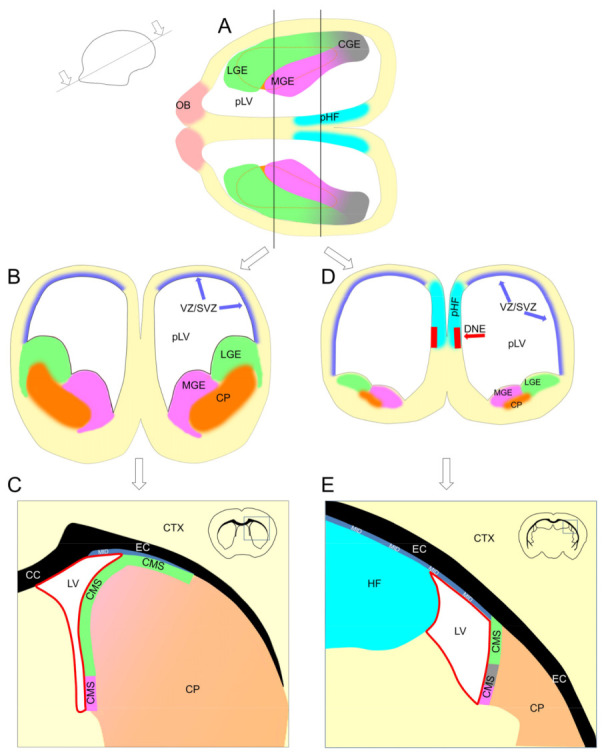
Formation of the pallial postnatal neurogenic (PPN) and subpallial postnatal neurogenic (SPPN) zones. (**A**,**B**,**D**) Embryonic mouse brain; (**C**,**E**) adult mouse brain. Formation of the PPN zone in the neocortex and archicortex. In the neocortex (CTX): the majority of neocortical excitatory neurons are produced in the pallial ventricular/subventricular zone (VZ/SVZ) located in the walls of the primordial lateral ventricle (pLV) (**B**,**D**). In the postnatal brain, the VZ disappears and is replaced by the ependyma (shown as a red line (**C**,**E**)), and the SVZ converts into the midlayer (MID), a thin layer of proliferating cells located under the external capsule (EC) (shown as a blue ribbon embossed with “MID” (**C**,**E**)). In the anterior part of the adult brain, the MID is located between the caudoputamen (CP) and EC (**C**), because the pLV in this part of the brain collapses due to the growth of the CP. In the posterior part of the brain, the pLV collapses due to the growth of the hippocampal formation (HF), and the MID becomes located between the CP and HF (**E**). Only a small fraction of the MID remains in the dorsal wall of the lateral ventricle (LV) (**C**,**E**). In the archicortex: the dentate neuroepithelium (DNE) is located in the septal wall of the pLV in the primordial hippocampal formation (pHF). In the postnatal brain, the DNE transforms into the subgranular zone (SGZ) in the HF (not shown). Formation of the SPPN zone: in the embryonic brain, the ganglionic eminences (medial (MGE), lateral (LGE), and caudal (CGE)) are located in the subpallium, in the walls of developing caudoputamen (CP) (**A**,**B**,**D**). They produce neuronal precursors for the limbic system and interneuronal precursors for the cortex and olfactory bulbs. In the postnatal brain, the ganglionic eminences transform into a layer of actively proliferated cells, the caudate migratory stream (CMS), located in the dorsal and medial walls of the CP (**C**,**E**). The part of the CMS located in the medial CP wall and facing the LV is often viewed as a separate neurogenic zone—the subependimal zone (SEZ). The growth of the CP during brain development leads to a partial collapse of the pLV, and the CMS in the dorsal CP wall becomes located between the CP and EC where it comes in contact with the MID (**C**).

The telencephalon development is supported by two principal zones of neurogenesis. One is the subpallial ganglionic eminences located in the walls of developing CP (Figure 10B,D), and another is the pallial ventricular/subventricular zone (VZ/SVZ) located in the lateral ventricle walls [13] (Figure 10B,D).

The ganglionic eminences produce neuronal precursors for the limbic system and interneuronal precursors for the cortex and olfactory bulbs. In the postnatal brain, the ganglionic eminences (Figure 10A,B,D) transform into a layer of actively proliferated cells located in the dorsal and medial walls of the CP (Figure 8 and Figure 10C,E). This layer, the CMS, includes the SEZ that corresponds to the CMS part located in the lateral walls of the lateral ventricle. The CMS in 10-day-old mice is still continuing to produce interneuronal precursors for the cortex [14]. However, in older mice, the CMS appears to produce precursors exclusively for the olfactory bulb interneurons [15,16,17]. The RMS connects the CMS with the olfactory bulbs and, therefore, can be combined in one compartment with it. We showed previously that neurogenesis can be detected in all parts of the CMS and RMS [12]. Thus, cell proliferation in the CMS and RMS is the direct continuation of developmental neurogenesis in the subpallium. We will refer to the CMS and RMS as the subpallial postnatal neurogenic (SPPN) zone.

The VZ/SVZ zone is located in the pallial walls facing the lateral ventricles [13] (Figure 10B,D). It produces precursors for cortical excitatory neurons and some interneurons. In the postnatal brain, the VZ disappears and is replaced by the ependyma (Figure 10C,E). At the same time, the SVZ in the neocortex converts into a thin layer of proliferating cells. This layer, the midlayer (Figure 8), is located between the hippocampal formation (HF) and EC or between the CP and EC due to collapse of the primordial lateral ventricles (pLV) in the postnatal brain (Figure 10C,E). Only a small fraction of the midlayer remains in the dorsal walls of the lateral ventricles (Figure 10C,E). In the archicortex, another part of the SVZ, originated from the dentate neuroepithelium (Figure 10D), converts into a thin layer of proliferating cells, the SGZ, located on the inner surface of the dentate gyrus (DG). Thus, the midlayer and SGZ can be combined in a single compartment not only on the basis of the similar density of proliferating cells but because of their common origin, the pallial SVZ, as well. We showed earlier that neurogenesis can be detected in all parts of the midlayer and SGZ [12]. Thus, cell proliferation in the midlayer and SGZ is the direct continuation of developmental neurogenesis in the pallial SVZ. We will refer to this compartment as the pallial postnatal neurogenic (PPN) zone.

The third proliferative compartment in the mouse brain likely corresponds to the postnatal production of oligodendrocytes in some white matter bundles in the telencephalon (Figure 7). The most prominent among these bundles are the CC and inner capsule (Figure 7). The fourth proliferative compartment corresponds to the postnatal production of precursors for renewal of microglia [18], cells of blood vessels, and oligodendrocytes [19,20,21] outside of the third compartment. The production of these precursors is supported by the adult stem cells and continues at a steady rate during the entire mouse lifespan (Figure 5).

The PPN and SPPN zones are thin layers with proliferating cells that can be easily identified on images of brain sections. Therefore, we can very accurately count the number of proliferating cells located in these zones. The neural progenitors account for about 98% of all proliferating cells in the SGZ (Figure 1F in [22]). There is no evidence that in other neurogenic zones the non-neurogenic precursors are produced in any substantial numbers. Thus, by estimating the number of new cells produced in the PPN and SPPN zones or their parts, we practically estimate the number of produced neural precursors. In contrast, we cannot estimate how many precursors for macroglia, blood vessel cells, oligodendrocytes, and other cells are produced in the third and fourth proliferative compartments. The production of these precursors is distributed throughout the entire brain and they can be only distinguished with the use of specific markers. Moreover, the difference in density of proliferating cells in the third and fourth proliferative compartments is not significant enough for their clear separation. Therefore, we can only estimate the number of new cells produced in the third and fourth proliferative compartments together.

We estimated that the SPPN and PPN zones produce about 25 million new cells (Table 2). This is sufficient to replace all 25 million neurons in the telencephalon, which has 18 million excitatory and 7 million inhibitory neurons [23]. However, this regenerative capacity is distributed very unevenly. The SPPN zone produces about 21.5 million precursors (Table 2) that move into the olfactory bulbs to supplement 600,000 olfactory interneurons [23]. Such amount of precursors is sufficient to replace all olfactory interneurons 36 times. The full replacement can be achieved in just 6–7 days in 2-month-old mice and in about 70 days in 30-month-old mice. On the whole, the SPPN zone produces about 85% of all neural precursors in the postnatal mouse brain. All these precursors are used to supplement or replace interneurons in the olfactory bulbs which constitute only 2.4% of all neurons in the telencephalon [23].

The PPN zone in the archicortex (the SGZ) produces about 0.8 million precursors (Table 2) for the DG granule cells. This amount is sufficient for the replacement of the majority of 1 million granular cells in the DG [23]. On the whole, the SGZ produces about 3% of all neural precursors in the postnatal brain, which are used to supplement 4% of all neurons in the telencephalon [23]. The PPN zone in the neocortex (in the midlayer that is also called the subcallosal zone [24]) produces about 2.7 million precursors or 12% of all precursors (Table 2). Neurogenesis in the midlayer is not well characterized. There are only a few publications about this neurogenic zone. They reported the production of oligodendrocytes [24] and neurons [25] in this zone. Therefore, it remains to be studied what is happening with the precursors produced in the midlayer.

There are about 21 million oligodendrocytes, 12 million microglia [23], and 7 million blood vessel cells [26] in the mouse brain. We estimated that about 31 million new precursors for these cells are produced in the third and fourth proliferative compartments between the ages of 2 and 30 months. This number is not sufficient for the replacement of all these cells even once during the entire mouse life. However, with our experimental approach, we cannot estimate the replacement rate of any specific group of cells in the mouse brain. Thus, there is a possibility that some kinds of cells can be renewed several times and others to a much smaller extent if at all. 

We estimated the production of new cells on the assumption that the S phase of the cell cycle lasts 12 h. Therefore, different lengths of the S phase in some proliferating cells should affect our estimates. Nevertheless, the production of precursors in neurogenic zones appears to be sufficient for substantial regeneration of lost neurons or repairing damage inflicted to the brain by trauma or neurodegenerative diseases. We need only to reprogram produced neural precursors and they will be able to replace not only granular cells and olfactory interneurons but other types of neurons in the brain as well. However, two major obstacles restrict this approach. The first obstacle is that neural stem cells have a very limited proliferative and self-renewing capacity. The second obstacle is that neurons cannot be replaced.

The first obstacle: The SGZ is the most thoroughly studied neurogenic zone in the postnatal mouse brain. Previous studies showed that the SGZ contains unusual stem cells which can be characterized as “disposable stem cells” [22] or “depleting stem cells” [27]. These cells divide only 2–3 times and produce on average 5–6 progeny before converting into somatic cells (Figure 1J in [27]). Even “long-term self-renewing stem cells” representing a small fraction of proliferating cells in the SGZ divide not more than 4 times and produce not more than 20 progenies (Figures 1J and 2F,G; Supplementary Materials in [27]). “Self-renewing stem cells” do not actually renew. Half of these cells convert into cells of other types or disappear within two weeks or less after going through a cell division (calculated on the basis of the lineage trees in Supplementary Figure 1 in [27]), and only about 10% of them remain after 30 days (Figure 1L in [28]). Consequently, the production of neural precursors never stabilizes and continues to decline progressively (Figure 2 in [3]) (Figure 5A) due to the disappearing population of “self-renewing stem cells” (Figure 1B in [28]). Additional discussion can be found in [29]. The continued decline of neural precursor production in other neurogenic zones (Figure 5A and Figure 9A) shows that neural stem cells in these compartments also have limited proliferative and self-renewing capacity.

The second obstacle: The tissue renewal in the adult mouse skin, blood, intestinal epithelia is attained by the replacement of dead or damaged cells on new ones. New cells assume the same role and occupy the same place as the lost ones allowing a tissue to maintain its functionality over time. However, it is impossible to replace a neuron that was lost or damaged. The role of neurons is to transmit and modify information. Each neuron in the brain forms synapses with a particular set of other neurons. These synapses allow each neuron to perform its unique function in processing information [30]. Therefore, a new neuron must restore all synapses of the lost one. However, new neurons are added to different locations from the lost neurons and form connections with different sets of neurons. Therefore, the memories, learned skills and cognitive abilities compromised by the neuronal loss cannot be simply restored by the addition of new neurons.

Thus, the production of neural progenitors in the postnatal brain cannot be sustained, especially at old age, when progenitors are needed the most for brain renewal and repair. In addition, new neurons differentiated from these progenitors cannot replace lost or damaged neurons and restore their functions. Well then, what can be the purpose of neural progenitor production in the postnatal brain? The answer to this question is straightforward—the continuation of brain development.

All processes in the mouse body can be classified as developmental, adult, and aging processes. The aging processes are characterized by the decrease of functional fitness and cell loss in organs and tissues. Postnatal neurogenesis does not fit this profile. The developmental and adult processes might require the addition of new cells, however with different purposes. New cells in the adult processes are produced to replace lost and damaged cells for renewing organs or tissues. At the same time, new cells in the developmental processes are produced to increase the functional capacity of organs or tissues.

Earlier we analyzed the postnatal hippocampal neurogenesis (the PPN in the archicortex) and concluded that it has all defining features of the late developmental process. This process occurs at the end of the organ and tissue development. The most apparent features of such a process are the decline of precursor production with time and programmed cell death of the majority of produced precursors [29]. The PPN in the neocortex clearly shows both these features. The production of precursors in the midlayer declines steadily with age (Figure 9A) and the majority of produced precursors undergo programmed cell death [25]. The SPPN also declines with age (Figure 9A) and only a very small fraction of progenitors produced by the SPPN are incorporated into the olfactory bulbs as interneurons. Using our data, we have calculated that approximately 3.6 million progenitors are produced by the SPPN in the mouse brain between the ages of 2 and 4 months (Table 1). At the same time, Imayoshi et al. reported that progenitors produced between the ages of 2 and 4 months account for only 20% of interneurons in the olfactory bulbs. That is about 120 thousand interneurons [23]. The measured number in the study was about 10% but the labeling efficiency in the experiment was about 50–60% (Figure 2P in [31]). It means that only 1 out of every 30 produced progenitors is incorporated into the olfactory bulbs. Therefore, the PPN and SPPN clearly display defining features of the late developmental process. The corresponding features in the adult processes are unmistakably different from the PPN and SPPN features. The adult processes are characterized by the steady-state production of precursors and their survival.

We have shown that postnatal neurogenesis continues in the remnants of all main developmental neurogenic zones in the telencephalon. As a result, it might retain a capacity to produce neural precursors for any part of the telencephalon in the postnatal brain. The reports on postnatal neurogenesis in the neocortex, striatum, amygdala, piriform cortex (reviewed in [32,33]) support this notion. The real extent of postnatal neurogenesis is still under investigation and we should not exclude a possibility that it might be continued in any part of the telencephalon.

The transition from the developmental to the adult stage is associated with the establishment of a niche with adult stem cells producing precursors for tissue renewal. Adult stem cells also provide a capacity for tissue and organ repair after their damage [34]. The mouse brain, as brains of other mammals including humans, continues its development postnatally. Consequently, we may hypothesize, the brain could not establish a niche with adult neural stem cells and gain a capacity for neuronal renewal and brain repair similar to other adult tissues. Instead, embryonic neurogenic zones transit into the postnatal brain and continue to produce neuronal precursors for brain development. Therefore, the brain can achieve regenerative capacity only via the continued development, which allows the brain to rectify damage in one place by adding neurons in another place. Such regeneration can be viewed as compensatory regeneration instead of the direct regeneration via replacement of lost and damaged neurons. Compensatory regeneration cannot be as efficient as direct regeneration in skin, blood, and other adult tissues. The low efficiency of compensatory regeneration explains the very limited ability of the postnatal brain to regenerate after neuronal loss or trauma even in the presence of robust production of neural precursors in the brain.

The density and number of proliferating cells appear to be maintained at the same level in all parts of the mouse brain, except for the telencephalon, between the ages of 2 and 30 months (Table 1, Figure 2 and Figure 7). We have not found any aggregations or gradients of proliferating cells in these parts of the brain that might indicate a continuation of development or modification of the cellular structure. On the whole, our results show that the cellular organization of the mouse brain, except the telencephalon, stabilizes and becomes mature by the age of 2 months.

The telencephalon, in contrast, shows a continuation of development that extends up to the age of 30 months. We identified three compartments in the telencephalon that have a density of proliferating cells higher than in surrounding parts of the brain. One of them includes the CC, inner capsule, and some other major white matter bundles in the telencephalon. The increased proliferation in this compartment can be detected at the ages of 2 and 4 months (Figure 7) showing that the cellular structure of these white matter bundles requires longer development and becomes mature only between the ages of 4 and 8 months. Two other compartments correspond to the PPN and SPPN zones. These zones are the remnants of major neurogenic zones operating in the embryonic telencephalon. The production of neural precursors in these zones substantially decreases with age (Figure 5A and Figure 9A, Table 1). However, even in a 30-month-old mouse, about 9 thousand new precursors are produced a day in the PPN and SPPN zones (Table 1). The production of precursors at such a rate during one month is sufficient for the replacement of 1% of all neurons in the telencephalon. The continuation of neural precursor production even in very old mice shows that the cellular structure of the telencephalon never completely matures and continues to change during the entire mouse life.

## 4. Materials and Methods

### 4.1. Animals and Tissue Collection

All experiments with mice, including euthanasia, meet The American Veterinary Medical Association (AVMA) guidelines and were conducted following the National Institutes of Health (NIH) and international guidelines and with veterinarian supervision. All experimental procedures were approved by The Institutional Animal Care and Use Committee (Department of Veterans Affairs, ENRM VA Hospital IACUC, Protocol SE-08-13-96).

C57BL/6J male mice were obtained from The Jackson Laboratory (Bar Harbor, ME, USA). The two mice per age group are listed in Table 1. We used a single injection of 5-Ethynyl-2′-deoxyuridine (EdU) to label proliferating cells. EdU staining uses small organic molecules and can be performed in under one hour [35]. We have previously shown that EdU could be stained through the entire thickness of 50 µm sections of the mouse brain [12]. Mice were injected intraperitoneally with a dose of 50 mg/kg (Thermo Fisher Scientific, Waltham, MA, USA). An injection dose above 50 mg/kg does not result in brighter staining of EdU-labeled cells or in an increase in the number of detected proliferating cells [35]. The labeling of proliferating cells for 2 h or longer resulted in the appearance of divided labeled cells in the mouse brain [12]. To avoid this complication, mice were euthanized one hour after EdU injection, transcardially perfused first with 20 mL of cold phosphate-buffered saline (PBS, pH 7.4) with 10 U/mL of heparin and then with 120 mL of cold PBS with 4% formaldehyde. Brains were extracted, incubated in PBS with 4% formaldehyde at + 4 °C for a day, and then transferred into 100 mM phosphate buffer, pH 7.4, with 20% of glycerol and 2% of Dimethylsulfoxide (DMSO) for at least two days before cutting.

### 4.2. Immunohistochemistry

Mouse brains were cut serially at 50 μm through the entire extent of the brain using a freezing sledge microtome. Sections were collected in a 24-well plate with wells filled with tris-buffered saline (TBS, pH 7.5). Transverse sectioning was used because the shape of the section is better maintained during the staining and mounting procedures which simplified the virtual reconstruction using the images of all sections of the mouse brain. Sections were first permeabilized using free-float incubation in a tris-buffered saline (TBS) solution with 0.5% triton-X100 for one hour at room temperature on a rocker table with gentle agitation. Sections were then transferred directly into the EdU Alexa 647 staining solution containing EdU staining buffer, CuSO4, Alexa Fluor 647 azide, and ascorbic acid in a proportion recommended by the manufacturer (Thermo Fisher Scientific, catalog# C10340) and incubated in the dark for one hour. Sections were then washed well in phosphate-buffered saline (PBS) (3 × 10 min), and all sections from each well were mounted on one gelatin-coated microscope slide (25 mm × 75 mm) and air-dried. Slides were coverslipped using a 5% propyl gallate/glycerol mounting media with 4′,6-diamidino-2-phenylindole (DAPI) and sealed with black nail polish.

### 4.3. Microscopy and Image Analysis

Image acquisition and analysis were performed as described previously [12]. Each microscope slide was scanned using the Zeiss AxioImagerZ2 microscope with an EC Plan-NEOFLUAR 5×/0.16 objective (Carl Zeiss AG, Oberkochen, Germany). We used a 5× objective for image acquisition because its focal depth (54.84 µm at 690 nm) exceeds the thickness of brain sections (after air drying, mounted 50 μm brain sections become approximately 25–27 μm thick) and the spatial resolution (1.29 µm × 1.29 µm per pixel) is sufficient for unambiguous detection of EdU-labeled nuclei. Each scan produced a 16-bit composite image of the entire microscope slide consisting of 600 individual images. Composite images were stitched, images for individual sections were cut out, arranged in the order according to their position in the brain, and manually registered [12]. We counted the number of all EdU-labeled nuclei in all brain sections using the Find Maxima Process with the tolerance parameter set to 1500 (Fiji, an open source platform for biological image analysis, release Madison, 7 March 2011, https://fiji.sc/, [36]) and obtained coordinates for each EdU-labeled nucleus. The tolerance parameter was set to 1500 because this allowed us to automatically identify all visually detectable EdU-labeled nuclei and at the same time had a low rate of false-positive identifications that we manually removed. To distinguish proliferating cells located in the MPZ, SGZ, and other parts of the mouse brain we manually selected all EdU-labeled nuclei located in these structures. We selected and counted EdU-labeled nuclei on all mouse brain sections.

### 4.4. Data/Statistical Analysis

Microsoft Excel was used for all data analysis and table and chart preparation.

To calculate the volume number density for each EdU-labeled nucleus in the SGZ, we calculated the distances between each EdU-labeled nucleus and all other EdU-labeled nuclei in the SVZ using the nuclei coordinates and then counted how many of them were located closer than 200 μm to this nucleus.

To calculate the number of new cells produced in the mouse brain we made an assumption that the S phase in all proliferating cells is 12 h long and that the change of proliferating cell number between analyzed time points is linear. To calculate the number of new cells produced between two analyzed points we used a formula S = (A + B) × C where S is the number of produced new cells; A is the number of proliferating cells detected at the beginning of the analyzed period; B is the number of proliferating cells detected at the end of the analyzed period, and C is the number of days in the analyzed period.

Statistical analyses were performed using a one-way analysis of variance (ANOVA) with significance set at *p*  <  0.05. The preliminary analysis shows that age has an extremely large effect on the number of proliferating cells in the neurogenic zones. In such circumstances, a *t*-test can be reliably applied to evaluate the significance of the difference between measurements with sample size *n*  =  2 [37].

### 4.5. Figure Preparation

Microsoft PowerPoint and Adobe Photoshop were used for figure preparation.

## Figures and Tables

**Table 1:** Age-related decrease in the number of proliferating cells (NPC) and average volume number density (AVND) of proliferating cells located in the mouse brain and its compartments.

Mouse	Mouse Age	Mouse Brain		Mouse Brain						MPZ						CMS			
				MPZ		SGZ		Other		CMS		RMS		Midlayer		Lateral Walls (SEZ)		Other Parts of CMS	
		NPC	AVND	NPC	AVND	NPC	AVND	NPC	AVND	NPC	AVND	NPC	AVND	NPC	AVND	NPC	AVND	NPC	AVND
2M-A	2 Months	68,010	193.3	45,524	283.7	2732	45.5	19,754	6.4	30,607	357.3	3559	323.9	11,358	72.7	14,087	272.2	16,520	429.9
2M-B	2 Months	67,636	173.7	44,505	259.5	2570	40.5	20,561	6.1	29,571	330.0	3234	287.5	11,700	73.5	13,343	270.5	16,228	378.8
4M-A	4 Months	52,489	127.4	32,030	205.3	1554	23.7	18,905	3.9	23,019	241.9	3366	239.4	5645	36.1	11,729	196.8	11,290	288.6
4M-B	4 Months	49,359	137.5	30,425	219.6	1581	25.6	17,353	4.8	22,012	254.9	3115	283.5	5298	35.3	10,184	193.6	11,828	307.7
8M-A	8 Months	42,711	82.5	19,376	176.6	625	12.2	22,710	4.9	14,881	184.1	3021	220.0	1474	11.1	8010	159.1	6871	210.7
8M-B	8 Months	40,904	76.4	18,901	160.7	613	12.8	21,390	4.4	14,969	170.5	2433	191.8	1499	11.4	7662	145.5	7307	196.7
12M-A	12 Months	33,200	48.4	12,869	119.8	325	6.7	20,006	3.8	10,057	120.2	2136	153.5	676	6.3	5926	107.2	4131	139.0
12M-B	12 Months	30,453	45.2	11,632	112.3	314	8.6	18,507	3.8	9974	118.4	1067	113.8	591	6.3	6255	106.7	3719	138.1
30M-A	30 Months	20,269	18.5	4877	64.9	70	2.7	15,322	4.2	3970	74.4	446	42.8	461	3.6	3337	76.4	633	63.9
30M-B	30 Months	20,215	13.9	4289	51.1	69	2.1	15,857	4.0	3592	58.5	306	25.8	391	3.4	2644	57.9	948	60.0

**Table 2:** Estimated numbers of new cells produced in the mouse brain and its compartments between ages 2 and 30 months.

		Mouse Brain			MPZ			CMS	
	Mouse Brain	MPZ	SGZ	Other	CMS	RMS	Midlayer	Lateral Walls	Other Parts of CMS
Mouse A	56,766,197	24,194,054	838,056	31,734,087	18,286,018	3,186,623	2,721,413	10,597,952	7,688,066
Mouse B	53,900,275	22,582,754	822,565	30,494,956	17,800,537	2,192,255	2,589,962	10,033,591	7,766,946
Average	55,333,236	23,388,404	830,311	31,114,522	18,043,278	2,689,439	2,655,688	10,315,772	7,727,506
STDEV	2026,513	1139,361	10,954	876,198	343,287	703,124	92,950	399,063	55,777

## Data Availability

All data generated or analyzed during this study are included in this published article.
